# Supplementary material for: Risk and protective factors for incidents of intimate partner violence among active-duty military personnel
Source: PLoS One. 2026 Feb 24;21(2):e0333816. doi: 10.1371/journal.pone.0333816 (PMC12931802; doi:10.1371/journal.pone.0333816)
Supplement: S1 Table — Note. SE = standard error; FAP = Family Advocacy Program; PTSS = posttraumatic stress symptoms. Male group N = 40,106; Female group N = 14,561. *p < .05. **p < .01. ***p < .001. (DOCX) [file pone.0333816.s001.docx]

| Variable | | Standardized coefficient (SE) | |
| --- | --- | --- | --- |
|  | Covariates | | |
|  | | Male | Female |
| Age, years | | −0.15 (0.03)*** | −0.24 (0.07)** |
| Race/ethnicity (White) | |  |  |
| Black, Non-Hispanic | | 0.15 (0.02)*** | 0.18 (0.03)*** |
| Hispanic | | 0.02 (0.02) | 0.03 (0.05) |
| Other | | 0.05 (0.03) | 0.04 (0.06) |
| Marital status (married) | |  |  |
| Single, never married | | −0.27 (0.04)*** | −0.13 (0.05)* |
| Divorced/widowed/separated | | −0.02 (0.02) | −0.06 (0.04) |
| Service branch (Army) | |  |  |
| Navy | | −0.04 (0.03) | −0.08 (0.06) |
| Marine Corps | | −0.07 (0.03)** | −0.04 (0.07) |
| Air Force | | 0.02 (0.03) | 0.00 (0.06) |
| Any prior FAP incidents | | 0.09 (0.01)*** | 0.07 (0.03)* |
| Observation time | | −0.00 (0.00)*** | −0.00 (0.00) |
|  | Risk factors | | |
| PTSS re-experiencing | | 0.02 (0.00)*** | 0.02 (0.00)*** |
| PTSS avoidance | | 0.00 (0.00)* | 0.01 (0.00)*** |
| PTSS numbing | | 0.04 (0.01)*** | 0.03 (0.01)*** |
| PTSS hyperarousal | | 0.02 (0.00)*** | 0.02 (0.00)*** |
| PTSS negative affect | | 0.14 (0.03)*** | 0.13 (0.03)*** |
| Alcohol dependence | | 0.05 (0.02)* | 0.05 (0.02)* |
|  | Protective factors | | |
| Socioeconomic | | −0.27 (0.03)*** | −0.26 (0.03)*** |
| Psychosocial | | −0.02 (0.00)*** | −0.04 (0.01)*** |
| Physical health related | | −0.03 (0.01)*** | −0.02 (0.00)*** |
| Career satisfaction | | −0.01 (0.00)*** | −0.01 (0.00)*** |
