# Supplementary material for: Risk and protective factors for incidents of intimate partner violence among active-duty military personnel
Source: PLoS One. 2026 Feb 24;21(2):e0333816. doi: 10.1371/journal.pone.0333816 (PMC12931802; doi:10.1371/journal.pone.0333816)
Supplement: S2 Table — Note. Coeff = unstandardized coefficient; LLCI = 95% lower limit for confidence interval; ULCI = 95% upper limit for confidence interval; AD = alcohol dependence; IPV = intimate partner violence; NA = negative affect; CS = career satisfaction; HR = health-related protective factors. Confidence intervals are based on 1,000 bootstrapped samples. Bolded confidence intervals are non-significant (i.e., encompass zero). Male group N = 40,106; Female group N = 14,561. *p < .05. **p < .01. ***p < .001. (DOCX) [file pone.0333816.s002.docx]

|  | Male group | | | Female group | | | |
| --- | --- | --- | --- | --- | --- | --- | --- |
| Indirect effect | Coeff. | LLCI | ULCI | | Coeff. | LLCI | ULCI |
| Numbing 🡪 AD 🡪 IPV | .008 | .001 | .015 | | .005 | .001 | .010 |
| Numbing 🡪 NA 🡪 IPV | .065 | .040 | .089 | | .054 | .033 | .076 |
| Re-experiencing 🡪 AD 🡪 IPV | .002 | **.000** | **.001** | | .002 | **.000** | **.005** |
| Re-experiencing 🡪 NA 🡪 IPV | .036 | .022 | .050 | | .036 | .022 | .050 |
| Hyperarousal 🡪 AD 🡪 IPV | .001 | **.000** | **.004** | | .001 | **.000** | **.004** |
| Hyperarousal 🡪 NA 🡪 IPV | .046 | .028 | .064 | | .031 | .019 | .044 |
| Avoidance 🡪 AD 🡪 IPV | .003 | .001 | .007 | | .003 | .001 | .007 |
| Avoidance 🡪 NA 🡪 IPV | .002 | **−.002** | **.007** | | .008 | .003 | .017 |
| CS 🡪 AD 🡪 IPV | −.001 | **−.003** | **.000** | | −.001 | **−.003** | **.000** |
| CS 🡪 NA 🡪 IPV | −.012 | −.018 | −.008 | | −.012 | −.018 | −.008 |
| HR 🡪 NA 🡪 IPV | −.064 | −.087 | −.039 | | −.048 | −.066 | −.030 |
| Psychosocial 🡪 AD 🡪 IPV | −.007 | −.013 | −.001 | | −.013 | −.024 | −.002 |
| Psychosocial 🡪 NA 🡪 IPV | −.040 | −.056 | −.025 | | −.063 | −.087 | −.038 |
| Socioeconomic 🡪 NA 🡪 IPV | −.015 | −.021 | −.009 | | −.015 | −.021 | −.009 |
