# Supplementary material for: Risk and protective factors for incidents of intimate partner violence among active-duty military personnel
Source: PLoS One. 2026 Feb 24;21(2):e0333816. doi: 10.1371/journal.pone.0333816 (PMC12931802; doi:10.1371/journal.pone.0333816)
Supplement: Attachment — Submitted filename: Response to Revierwers 9Dec25.docx [file pone.0333816.s003.docx]

**PONE-D-25-34092 Revision**

**Itemized List of Responses**

Thank you for the constructive reviews we received in response to our article titled, “Risk and protective factors for incidents of intimate partner violence among active duty military personnel” (PONE-D-25-34092). We appreciate the encouragement we received regarding the relevance of this topic, the quality of the writing, the appropriateness of the analysis, and the quality of the data we have used.

Below we have itemized the feedback from our reviewers, as well as the general editorial comments we received with respect to PLOS ONE requirements. After each recommendation, we have described our responses and revisions highlighted in blue font.

**PLOSE ONE requirements:**

1. Please ensure that your manuscript meets PLOS ONE's style requirements, including those for file naming. The PLOS ONE style templates can be found at https://journals.plos.org/plosone/s/file?id=wjVg/PLOSOne_formatting_sample_main_body.pdf and https://journals.plos.org/plosone/s/file?id=ba62/PLOSOne_formatting_sample_title_authors_affiliations.pdf.

**We have reviewed the reference links and updated formatting, and figure file naming conventions to meet PLOSE ONE style requirements.**

2. Thank you for stating the following financial disclosure: “Defense Health Agency, U.S. Department of Defense.” Please state what role the funders took in the study. If the funders had no role, please state: "The funders had no role in study design, data collection and analysis, decision to publish, or preparation of the manuscript." If this statement is not correct you must amend it as needed. Please include this amended Role of Funder statement in your cover letter; we will change the online submission form on your behalf.

**The Defense Health Agency, our funding institution, was not involved in any aspects of this study. We have moved the suggested non-involvement statement up from the end of our disclaimer to immediately follows the sentence identifying the Defense Health Agency as the funder. We appreciate your assistance in further changing the online submission form to add this statement: "The funders had no role in study design, data collection and analysis, decision to publish, or preparation of the manuscript."**

3. In the online submission form, you indicated that your data is available only on request from a third party. Please note that your Data Availability Statement is currently missing [the name of the third party contact or institution / contact details for the third party, such as an email address or a link to where data requests can be made]. Please update your statement with the missing information.

**We have updated our data availability statement with a contact email and instructions regarding how to contact the Millennium Cohort Program to request permission for data extracts and collaborative research. We also have added the website link for submitting data requests to the Defense Manpower Data Center, which houses DoD personnel records and the FAP Central Registry data utilized in this report.**

4. Please ensure that you include a title page within your main document. You should list all authors and all affiliations as per our author instructions and clearly indicate the corresponding author.

**We have added the title page to our main document, listing all authors and affiliations per instruction. Dr. Stander is identified as corresponding author.**

5. If the reviewer comments include a recommendation to cite specific previously published works, please review and evaluate these publications to determine whether they are relevant and should be cited. There is no requirement to cite these works unless the editor has indicated otherwise.

**We have taken this direction under advisement in responding to reviewer feedback.**

**Reviewer 1:**

This study investigates modifiable risk and protective factors for intimate partner violence (IPV) perpetration among active duty U.S. military personnel, with the goal of informing prevention and intervention strategies. The authors employ a prospective path model using structural equation modeling (SEM), with IPV incidents—defined according to Department of Defense (DoD) criteria for psychological and physical abuse—as the primary outcome. Key risk factors identified include posttraumatic stress symptoms (PTSS), alcohol dependence, younger age, enlisted status, and prior IPV incidents. Protective factors are categorized into three domains: socioeconomic, psychosocial, and physical health.

6. Latent Variable Construction: Discriminant function analysis is not a conventional method for constructing latent variables in SEM. Confirmatory factor analysis (CFA) would be more standard and interpretable.

Methodological Justification: The rationale for selecting discriminant analysis over CFA should be clarified to help readers understand the methodological choices.

**We agree with the reviewer that use of discriminant function analysis is unconventional, and we do owe the reader better documentation for that choice. As suggested, we have now revised the analysis subsection of our methods to clarify that the risk and protective factors in our model are best conceptualized as composite-formative factors, in contrast to the more traditional reflective indicators of latent constructs estimated with CFA. We further explain that we expect the variables in each set/cluster to have a correlated synergy with a common impact on IPV. Therefore, our aim was to extract a factor representing the summary effect of each set of intercorrelated variables specifically on IPV. This is in line with the definition of composite-formative factors presented by Bollen (2017):**

**A set of observed variables (x’s) which are not assumed to have conceptual unity, (b) form an exact weighted linear composite (C), with (c) weights (w’s) that are typically estimated empirically. We use the term “composite-formative” to refer to this type of indicator.**

**Bollen, K. A. (2011). Evaluating effect, composite, and causal indicators in structural equation models. MIS Quarterly, 35(2), 359-372. https://doi.org/https://doi.org/10.2307/23044047**

7. Model Fit Reporting: The manuscript should report a broader range of SEM fit indices (e.g., RMSEA, CFI, TLI), which are standard in SEM reporting and would enhance transparency.

**RMSEA, CFI, and TLI fit indices are not optimal for logit models in SEM, because they assume continuous, multivariate normal data. However, the Logit model was selected because our outcome is dichotomous. In preparing our analytic plan, we spent time investigating the appropriate criteria to use in evaluating model fit under these conditions. We previously included a paragraph explaining how the AIC criteria functions in the analyses section of our methods. However, now we also have added a statement regarding why AIC was selected instead of the more traditional RMSEA, CFI, and TLI.**

8. Binary Outcome Modeling: While using a binary outcome (IPV) in SEM is valid, the manuscript lacks detail on whether robust estimators or logit link functions were used. This should be clarified.

**We did not use the logit link function or robust estimators originally. However, in this revision, we have redone our analyses to use robust estimation. We have added a statement in our methods to reflect this.**

**Shifting to the use of robust estimators made little difference in results, given coefficient estimates are identical between maximum likelihood estimation with and without robust standard errors. However, we have updated some p values due to this change; also, there was an association between re-experiencing and alcohol dependence that was only detected in our final model that is no longer significant with robust estimation. We have removed references to that association.**

9. Sensitivity Analysis: Effect size changes in the sensitivity analyses should be reported more explicitly to quantify the impact of model adjustments.

**We believe the wording of our presentation of the sensitivity analyses may have created confusion here. The change in effect sizes noted were specifically for the association between the modified health-related protective factor and the alternative PTSS negative affect factors. We have listed specific changes in those effect sizes within the sensitivity analysis section. Also, to avoid confusion and make it more evident that the referenced changes were limited to this relationship, we have revised the last sentence to specify more clearly that it was effect sizes “between these two constructs” that were impacted.**

10. Table 1 Reporting: More precise p-values should be provided in Table 1 to improve interpretability and reproducibility.

**At the reviewers recommendation, we have updated Table 1 to include precise p-values.**

**Reviewer 2:**

This paper described research into the relationship between PTSD and IPV, highlighting potentially modifiable risk factors for perpetration. While the methodology and findings were largely clear, I have queries about the conceptualization of the models in the main.

11. While I agree with some of the hypotheses presented, it would be useful for further discussion of them in the introduction to better support the development and thinking behind the hypothesized model, for example, fuller presentation of research of PTSD clusters and potential impacts on IPV or relationship tension and of negative affect. This would help highlight why direct pathways between PTSS symptoms and IPVA were not included and the hypothesis of full mediation pursued given what is known of PTSD clusters and IPVA.

**We appreciate the reviewer pointing out that our introduction was too thin in supporting the primary mediational hypothesis and prominence of hyperarousal as a predictor of IPV; we have added clarity in the introduction regarding the potential role of hyperarousal as a mediator.**

12. I also query the inclusion of workplace satisfaction within socioeconomic factors. While satisfaction may be related to SES, one can have low SES and high job satisfaction. There is likely other research to support the point being made about links between SES with IPVA, especially given recruitment practices for the military. Stith and others may fit better as psychosocial factors.

**We agree that this was not well conceptualized originally, and we believe the results of our measurement model provide empirical evidence for this. Based on a low factor loading for this variable, we did remove this variable from the SES factor in the process of conducting our analyses and we have entered it as a separate protective predictor in all final models. In order to be transparent about our research trajectory, we hope it is acceptable to continue to describe the original way we hypothesized this and how we came to model it separately in the paper. Also, note that we have clarified this further in our discussion and limitations.**

13. The discussion could include great reference to prior literature as well as an overview of the findings to better contextualize findings not only in terms of research and what is being added, supported or disputed, but what particular interventions could realistically be introduced?

**We edited the discussion to make the implications of our results for theory clearer. Also, as requested, we have added additional discussion regarding implications for targeted prevention, particularly related to modifiable factors like social support, as well as for tailored intervention.**

14. Some additional references needed in parts e.g., for the first line, page 6 line 116-118 regarding benefits of service etc.

**Thank you, we have added a reference in the location requested, as well as some in the discussion (see paragraph 6). These references similarly provide documentation for DoD programs and services, as well as advances in communication tools supporting military families.**

15. While not a major point, the large sample is extremely impressive but would not necessarily offset systematic under-reporting of IPVA within DOD systems, especially when used as the outcome of interest (and a prevalence of only 1% is very low). This is unfortunately one of those limitations that must be embraced without current ability to address within the dataset until more robust measures of IPVA can be included.

**We have added additional emphasis in our discussion and limitations regarding this weakness.**

16. It would be useful to include DOD definitions of IPVA which are not explicitly stated making it hard to determine what exactly may have been included if it was in fact reported.

**We would like to thank Reviewer 2 for pointing out this omission. Although we included information in our Methods and measures about the data collected by the DoD central registry to document IPV, we did not cite the DoD definition of domestic abuse. We have now added that citation.**

17. Finally, there is also the point that, while not a focus here, those with mental health problems are perhaps at greater risk of experiencing IPVA than of perpetrating it.

**We agree this is important and have now added discussion about this. Specifically, we now raise the issue of high rates of bi-directional IPV in military populations within the conclusion. We believe this also helps further address point #13 above.**
