# Supplementary material for: Risk and protective factors for incidents of intimate partner violence among active-duty military personnel
Source: PLoS One. 2026 Feb 24;21(2):e0333816. doi: 10.1371/journal.pone.0333816 (PMC12931802; doi:10.1371/journal.pone.0333816)
Supplement: Attachment — Submitted filename: Response to reviewers (26Jan26).docx [file pone.0333816.s004.docx]

**PONE-D-25-34092 Revision**

**Itemized List of Responses**

We appreciate the additional feedback we received in response to the resubmission of our article titled, “Risk and protective factors for incidents of intimate partner violence among active duty military personnel” (PONE-D-25-34092). Below we have itemized this feedback, along with our responses highlighted in blue font.

Reviewer #1: The revisions are comprehensive and directly respond to the methodological and reporting concerns. The manuscript now provides clearer justification for analytic choices, improved transparency in reporting, and enhanced clarity in sensitivity analyses.

**Thank you for this positive feedback. We are happy our revisions have improved this manuscript.**

Reviewer #2: Thank you to the authors for their considered responses to reviewer comments. The methods in particular are much clearer and the decision made in analyses more robustly justified. There are some remaining comments that would further clarify and strengthen the paper but these are very minor.

1. There are some statements in the intro and overview of prior research that still need references (e.g., first few sentences, top of page 7 re anger and hyperarousal, sleep problems, end line 132). While SES is important, a nod to considering the role of military cultural factors in IPVA (eg work of Deirdre MacManus et al in UK) would also be useful even if not captured within the data.

**We have added references in the places suggested. Also, we now have included a reference to the work of Deirdre MacManus at the end of the first paragraph of the introduction.**

2. I appreciate the inclusion of more discussion of issues of directionality and on the outcome measure itself. However, these should be earlier in the paper. The addition of the DOD definition is useful though it would be helpful for this to come earlier in the paper, or for a definition of IPVA to be added into the first paragraph and perhaps a note on any discrepancies between the two in the limitations. For example, it looks like the DOD definition doesn't include sexual IPVA (although this is mentioned later in the binary variable description) but wider IPVA definitions certainly would. The point on directionality could be made earlier also, methods perhaps, without undermining the paper as all data has limitations.

**We appreciate Reviewer 2’s suggestions of alternative strategies to improve presentation of the definition of IPV. We have followed the reviewers suggestion to add a definition of IPV in the first paragraph of the manuscript and then point out divergences between that definition and the DoD operationalization of domestic abuse both in our Methods (see Measures) and in our limitations.**

**With respect to the issue of bi-directional IPV, we believe bringing this up in the introduction of the paper would detract from the flow and potentially confuse readers, as this is not a focus in our analysis. However, in the methods where we describe the FAP Central Registry data, we now have clarified that information regarding both perpetration and victimization are available in the Central Registry. In the discussion, we also have added the fact that this paper only focused on risk and protective factors for IPV perpetration as a limitation. We further recommend future research in this area and cite a related publication we have done that does move into this topic in case the reader is interested. We hope this approach in addressing this is acceptable.**

3. The gender sub-analyses were interesting, but it would be useful to have an idea of how many women were in the dataset at this point.

**We have added the size of the subsample of women and the numbers with FAP reports by gender in the first paragraph of the results. We also have added these subgroup sample sizes in the figure and table notes where sex comparisons have been made. We agree this is important information to include.**
